# The Effect of Habitat on Insect Movements: Experimental Evidence from Wild-Caught Butterflies

**DOI:** 10.3390/insects14090737

**Published:** 2023-08-31

**Authors:** Matteo Marcantonio, Raluca Voda, Daniele Da Re, Quentin Igot, Roger L. H. Dennis, Aurélien Vielfaure, Sophie O. Vanwambeke, Caroline M. Nieberding

**Affiliations:** 1 Earth & Life Institute, University of Louvain (UCLouvain), Carnoy Building, Croix du sud 4-5, 1348 Louvain-la-Neuve, Belgium; vielfaure.aurelien@gmail.com (A.V.); caroline.nieberding@uclouvain.be (C.M.N.); 2 Centre for Earth and Climate Research, Earth & Life Institute, University of Louvain (UCLouvain), Place Louis Pasteur 3, Bâtiment Mercator, 1348 Louvain-la-Neuve, Belgium; daniele.dare@uclouvain.be (D.D.R.); sophie.vanwambeke@uclouvain.be (S.O.V.); 3 UK Centre for Ecology & Hydrology, Maclean Building, Benson Lane, Wallingford OX10 8BB, UK; rlhdennis@aol.com; 4 L’Institut Agro Dijon, 26, bd Docteur Petitjean-BP 87999, 21079 Dijon, France

**Keywords:** behavioural ecology, oviposition site selection, butterflies, habitat fragmentation, land-use changes, *Limenitis camilla*

## Abstract

**Simple Summary:**

Habitat loss profoundly influences animal behaviour, impacting the long-term viability of species. In this study, we explored the link between the flight capabilities of the woodland-specialist butterfly, *Limenitis camilla*, and the spatial-temporal connectivity of its main habitat—broadleaved forests. We examined the shifts in forest functional connectivity in Wallonia (Belgium) in recent decades, alongside the exploratory flight patterns of female *L. camilla*. Our findings revealed that butterflies from fragmented forests spent more time on movement-related behaviours than those from well-connected habitats. This indicates a potential evolutionary adaptation favouring behaviours that help locate suitable egg-laying sites in populations that have experienced habitat shrinkage and increased isolation over the past 20–40 generations.

**Abstract:**

There is broad evidence that the main driver of the ongoing biodiversity crisis is land-use change, which reduces and fragments habitats. The consequence of habitat fragmentation on behavioural responses of fitness-related traits in insects have been so far understudied. In herbivorous insects, oviposition-related behaviours determine access to larval food, and the fate of the next generation. We present a pilot study to assess differences in behaviours related to movement and oviposition in *Limenitis camilla* butterflies from Wallonia (Belgium), one of the most fragmented regions in Europe. We first quantified variation in functional habitat connectivity across Wallonia and found that fragmented habitats had more abundant, but less evenly distributed host plants of *L. camilla*. Secondly, we quantified the behaviours of field-caught *L. camilla* females originating from habitats with contrasted landscape connectivity in an outdoor experimental setting. We found differences in behaviours related to flight investment: butterflies from fragmented woodlands spent more time in departing flight, which we associated with dispersal, than butterflies from homogenous woodlands. Although results from this study should be interpreted with caution given the limited sample size, they provide valuable insights for the advancement of behavioural research that aims to assess the effects of global changes on insects.

## 1. Introduction

The rate of species extinction is higher than ever before due to human activities [1]. A recent global assessment of biodiversity and ecosystem services has shown that a quarter of all known species are threatened, whereas about one million are already facing extinction in the short term [2]. The current biodiversity crisis is mostly driven by land-use changes, which increase habitat loss and fragmentation [3], resulting in cascading negative effects on the biota [4]. Particularly susceptible to contemporary changes are insects, the most species-rich group of organisms on Earth [5]. Insects, including many butterfly species, are vulnerable to human-induced rapid environmental changes (“HIREC” hereafter) and especially to land-use changes, including modern industrial farming and forestry practices [6]. Butterflies are key bioindicators of habitat quality and flagship species for documenting the ongoing biodiversity crisis since their population and distribution changes have been monitored for decades [7]. Currently, about 19% of all European butterfly species are in the IUCN categories threatened or near threatened, with populations rapidly declining [7]. Extinction risk of European butterflies is highly associated with habitat loss and fragmentation, in particular due to impacts on larval host plants and/or adult habitats [8,9]. During their lifetime, butterflies depend on multiple habitats, which taken together form their “functional habitat” [10] and the degradation of one or more components of their functional habitat imposes important constraints on their population dynamics. Butterflies that rely on few larval host plants and have stricter habitat requirements were shown to be more at risk of extinction than species with a larger range of preferences [11], similar to findings in other insects (review [12], moths [13,14]).

HIREC has consequences for both the availability and configuration of suitable habitats and these changes may alter the organism-functional habitat relationships through changes in behaviour [15]. Behavioural changes affect in turn species interactions, population dynamics, evolutionary processes and biodiversity patterns [4,16]. How animals respond to HIREC, and how this influences population survival, is an area of growing research interest [17,18,19]. Yet, despite the vital links between environmental change, behaviour and population survival, the study of the interplay between behavioural phenotypes under HIREC is still at a very early stage. Behavioural change has the potential to mitigate or exacerbate the influence of environmental heterogeneity in the short time frame of HIREC [16,20] and has emerged as a possible major evolutionary mechanism for species to face HIREC, both at the theoretical [21] and the empirical level [22,23,24,25].

One documented behavioural change related to abrupt and rapid alterations of the environment is linked to movement [16]. Movement of an organism between different patches of its habitat relates to different life functions acting across multiple spatial and temporal scales, and the decision to move is based on the individual internal state, motion and navigation capacity and the external factors in the environment that affect its habitat [26,27]. Behavioural responses to fragmentation related to movement can vary across taxa: different bird species increase their habitat by also using suboptimal areas [28,29]; some large mammals manifest avoidance of large portions of their habitat range and prefer staying in familiar areas as a consequence of anthropogenic development [30,31]. Insects were shown to avoid dispersal as well: *Bombus veteranus* bumblebees preferred to stay inside fragmented patches rather than visiting more distant areas, thus reducing pollen dispersal and increasing inbreeding of the visited plants [32]. Reduced mobility was also documented in butterfly populations originating from fragmented landscapes: *Papilio machaon* and *Maculinea arion* had reduced thoracic size, which may be linked to a reduced flight capacity [33]. On the contrary, a heavier thorax that allowed better dispersal was found in isolated *Hesperia comma* populations [34]. Land-use intensification was also shown to favour generalist over specialist-associated traits in butterfly communities, including increased dispersal and longer distance flight [35]. An increase in the costs related to dispersal in fragmented landscapes was shown in *Proclossiana eunomia*—butterflies in fragmented landscapes displayed a straighter and longer flight [36].

In addition to movements, changes in reproductive behaviours in butterflies are crucial in small and fragmented patches of habitat, due to host plant availability. As habitats become increasingly smaller in space and fragmented, female butterflies may allocate more resources for longer flights to locate the host plants and less resources for egg production, which may result in a resource allocation trade-off between egg production and flight investment [37]. In herbivorous insects with limited mobility in the larval stage, such as butterflies, the site of oviposition is also the food source of the offspring, which makes the dispersal movements of the adults decisive for the selection of the oviposition site. Indeed, egg-laying choices in herbivorous insects have consequences on offspring growth [38], defence [39] and competition [40]. A study on the butterfly *Maniola jurtina* estimated gene flow as an indirect method to account for reproductive success and found that dispersal is higher in landscapes similar in structure to the species’ optimal habitat (grasslands), as opposed to arable lands and woodlands, indicating that dispersal through unsuitable habitats (i.e., the “landscape matrix”) can reduce gene flow and thus reproductive success [41]. *Pararge aegeria* butterflies from fragmented, agricultural landscapes, where host plants may be distributed more widely, laid fewer but larger eggs in comparison with butterflies from woodland landscapes [37]. Moreover, *P. aegeria* from agricultural habitats displayed a more exploratory behaviour related to oviposition site selection [42]. Conversely, butterflies of other insect species may employ other strategies, produce more eggs of a smaller size and spend less time searching for high-quality oviposition sites [43].

In this context, we addressed the question of whether habitat fragmentation has an effect on behavioural strategies related to oviposition site selection and other movements in the woodland specialist butterfly *Limenitis camilla* (Lepidoptera: Nymphalidae). We collected adult butterflies from woodland sites that have remained conserved and connected in the last five decades (i.e., “homogeneous habitats” hereafter), as well as from smaller, patchy woodland remnants (i.e., “fragmented habitats” hereafter) across Wallonia, Belgium. We quantified the activity of wild-caught butterflies in outdoor cross-shaped experimental cages, by focussing on flight activities in relation to site selection for oviposition, while controlling for the effect of abiotic factors such as temperature, humidity and insolation. Our overarching goal was to assess whether butterflies originating from homogenous vs. fragmented habitats showed behavioural differences related to movement and oviposition under ecologically relevant conditions. We answered the following specific questions:

- Does the level of spatial and temporal connectivity of *L. camilla* habitats vary across Wallonia? We expected (E1) that in this region there were areas with diverse habitat-change trajectories that could be pinpointed in space and time.
- Does host plant abundance and spatial distribution differ between homogenous vs. fragmented habitats? We expected (E2) that host plants were more abundant and more evenly distributed in homogenous compared to fragmented habitats.
- Do butterflies modify their behaviour due to the presence of the host plant in the experimental setup? We expected (E3) that butterflies spent relatively more time in the tunnel with the host plant than in the control tunnels, while accounting for abiotic factors that could have affected their movements in the tunnels.
- Do butterflies originating from fragmented vs. homogenous habitats differ in the time they allocate to different movement behaviours? We expected (E4) that individuals from fragmented habitats were more dispersive and spent less time navigating the tunnels in search of the host plant.

## 2. Material and Methods

### 2.1. The Model Species Limenitis camilla

*Limenitis camilla* is a widespread species in the Palearctic and it is also present across Belgium, where it is strictly associated with remnant woodland habitats. This species is locally common in southern areas of Belgium, and in the last decades populations have been increasing in abundance also in the north of the country [44]. *Limenitis camilla* is usually univoltine, flying from mid- or late June until mid-August and it occurs in moist broadleaved woodlands, where it is associated with open sunny areas in the tree vegetation cover [10,45]. In Wallonia, it oviposits on *Lonicera periclymenum* (Dipsacales: Caprifoliaceae) and *L. xylosteum* (Dipsacales: Caprifoliaceae) [44]. *Lonicera periclymenum* is a vine, which prefers acidic terrain and grows close to the ground in the interior of temperate oak forests, whereas in canopy gaps, forest fringes and wooded banks it is a flowering winding climber [46]. *Lonicera xylosteum* is a small shrub that occurs on well-drained and calcareous soils in beech-woods or open mixed woods [47]. *Limenitis camilla* prefers to oviposit on plants located in semi-shade or dappled light inside the forest or at their edges [47] and its larvae are not able to develop under full-sun conditions due to the sticky secretions of *Lonicera* leaf trichomes that hinder the construction of the larval hibernaculum [48].

### 2.2. Butterfly Occurrence Dataset

**Figure 1:**
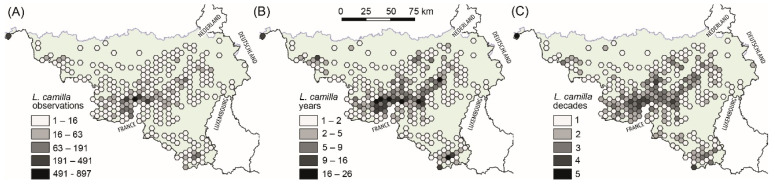
Maps with 5 km hexagonal grid reporting observations of *Limenitis camilla* in Wallonia from 1979 to 2019: (**A**) the total number of observations; (**B**) the number of years with at least one observations and (**C**) the number of decades with at least one observation.

Butterfly occurrences for Wallonia were provided by the Direction de la Nature et de l’Eau (DNE-DEMNA-SPW-ARNE et collaborators, agreement contract of sharing data n°CMDD1351). The database comprised 1825 geo-referenced *L. camilla* observations spanning from 1970 to 2019. We aggregated *L. camilla* occurrences by using a 5 km hexagonal grid, considering the number of observations, the number of years with observations and the number of decades with observations (Figure 1). Thus, we selected areas in Wallonia where *L. camilla* populations are both currently abundant and stable in time.

### 2.3. Functional Habitat Connectivity and Selection of Sampling Areas

#### Land Use Analysis

**Figure 2:**
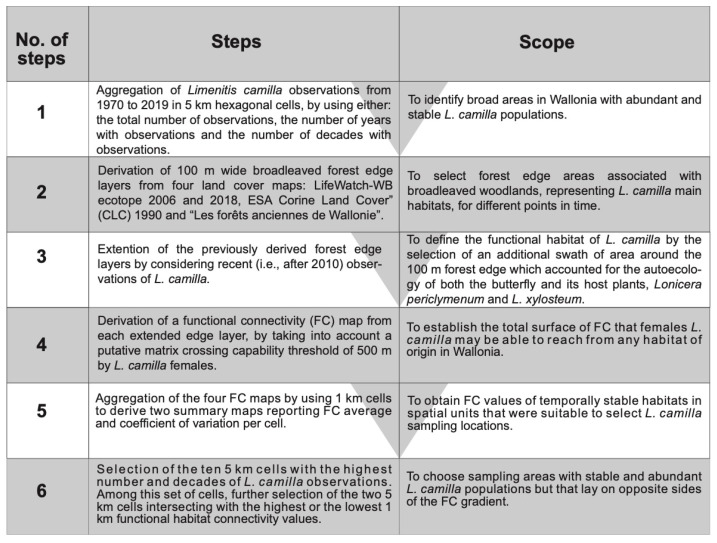
Steps followed to define the functional habitat for *L. camilla* and to choose the collection sites with stable and abundant *L. camilla* populations in Wallonia.

We selected *L. camilla* populations originating from broadleaved woodlands with different degrees of spatial connectivity and temporal stability in Wallonia. To pinpoint these forests, we used standard, open-access land cover datasets (Figure 2): “LifeWatch-WB ecotope” (ECO) for the years 2006 and 2018 (http://maps.elie.ucl.ac.be/lifewatch/ecotopes.html (accessed on 3 September 2022), “ESA Corine Land Cover” (CLC) 1990 (https://land.copernicus.eu/pan-european/corine-land-cover/clc-1990/# (accessed on 3 September 2022)) and “Les forêts anciennes de Wallonie” (Ancient Forests), representing the estimated forest coverage in Wallonia during the 18th century and based on the “Ferraris map” (https://opac.kbr.be/LIBRARY/doc/SYRACUSE/16992733 (accessed on 3 September 2022)). Each of these spatial layers was imported in the software GRASS GIS v7.9 [49] and subset by selecting only land cover classes corresponding to broadleaved forests, preferred by *L. camilla*. This land use category corresponded to class 60 in ECO 2018, 2006 and CLC 1990 and to “boisement feuillu” and “forêt ancienne subnaturelle” in the Ancient Forests database. These four datasets were first transformed in binary raster data (1 = forest, 0 = matrix) with a spatial resolution of 10 m using bilinear interpolation and were then used to derive matrix-edge-core maps (MAC) using the open-access software LS Metrics [50]. For this step, we defined an “edge” as the 100 m swath (or 10 pixels) area between the matrix and the core of a broadleaved forest patch [51]. As a further step, we derived the distances between *L. camilla* observations and the edge of forest habitats (from ECO 2018) and compared it with all other butterfly observations in our dataset. A two-sample *t*-test showed that *L. camilla* was found at significantly shorter (interior) distances from forest edges (mean = −34 m, SD = 106) than all other butterflies’ observations (mean = 51 m, SD = 153 m; t(283) = 12.4, *p* < 0.001). We consolidated this information by using the interquartile range (IQ) of *L. camilla* distance-distribution (IQ range = −86, +11 m) in order to select an additional area around the 50 m forest edge which accounted for the ecology of both *L. camilla* and its host plants *Lonicera periclymenum* and *L. xylosteum,* while also considering the spatial distribution of recent butterfly observations. We defined this “extended forest edge” (EFE) swath as the functional habitat of *L. camilla*, or the habitat wherein the species is able to complete its life cycle—thus including mating, breeding and foraging habitats. We extracted pixels belonging to this category from each of the four spatial layers in separate binary raster maps (EFE maps [10,52]). These “functional habitat” maps were used as input in LS Metrics to derive functional connectivity (FC) maps, by considering a crossing capability threshold of 500 m. This crossing capability can be assumed to approximate the maximum Euclidean distance that *L. camilla* butterflies fly from a functional habitat patch to reach another functional patch (see for example [53]). In addition, this figure is in the range of the dispersal capability of larger and dispersive butterfly species (e.g., *Gonepteryx rhamni*, whose dispersal ability between habitat patches is >1 km) and smaller and more sedentary species (e.g., *Leptidea sinapis*; 300 m). The FC maps thus had pixel values corresponding to the functional habitat area in hectares available for a putative dispersing *L. camilla* located in that pixel. We derived two summary FC maps reporting the temporal average and coefficient of variation by considering the four FC maps. As a final step, these two maps were aggregated using a hexagonal 1 km grid which allowed us to obtain spatial units that could be readily used as reference sampling locations. Thus, the final 1 km maps represented the average functional habitat connectivity and its variability over the four considered time points (18th century, 1990, 2006 and 2018).

### 2.4. Sampling Populations of L. camilla and Its Host Plant L. periclymenum

We sorted the 5 km hexagonal cells (see Figure 1) by using the highest number of occurrences, and then decades and years with observations for Wallonia as partial sorting criteria. Afterwards, we selected two 5 km cells with, respectively, the highest and lowest 1 km functional habitat connectivity values. These 1 km cells represented the areas where we collected *L. camilla* (from the end of June to the beginning of August; thus, we assumed that all butterflies were collected after mating) and surveyed for the presence and abundance of its host plants. We chose to assess presence and abundance of the host plant inside or in the vicinity of the same 1 km hexagonal cell where *L. camilla* were also sampled. Thus, we selected only EFE habitats inside each 1 km cell where butterflies were collected and drew ten random points inside each of these areas using QGIS v3.16 tool “Random points inside polygons”. For the random point draw, we added a constraint of a minimum 200 m distance between points to avoid pseudo-replication due to spatial autocorrelation. A subset of these points was used as central coordinates to build 20 × 20 m vegetation surveys aligned along cardinal directions. We divided the plot in 5 × 5 m quadrants, where we recorded the presence and total number of host plants stems (limiting the count to a maximum of 25 stems per plot due to time constraints), in order to have more resolution for assessing host plant spatial distribution.

### 2.5. Behavioural Experiments and Data Analyses

Upon field collection, butterflies were kept in individual 11 × 11 × 14 cm transparent plastic boxes placed inside Sanyo incubators under constant conditions (temperature day/night: 22 °C/16 °C; relative humidity: 60%; photo-period light/dark: 2 h/22 h under solar light spectrum simulating lamps; Philips HPI-T Plus 400W/645) for at least 24 h before behavioural experiments. These conditions were intended to reduce activity and thus wing wear, and to stimulate activity and oviposition during behavioural trials. All butterflies had access to 20% sugar solution and water ad libitum during this phase.

**Figure 3:**
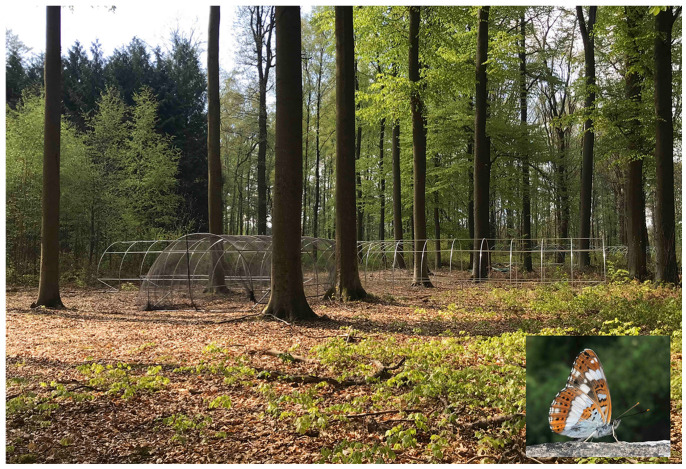
An outdoor flight setup consisting of four cross-shaped greenhouse aluminium tunnels covered with insect mesh, placed in “Bois de Lauzelle” forest, an experimental site belonging to UCLouvain, and an inset representing the study species *Limenitis camilla*. Photos: R. Voda.

We conducted behavioural experiments in outdoor flight arenas, which consisted of four cross-shaped greenhouse aluminium tunnels, each 8 × 3 × 2 m covered with a thin black insect mesh and placed in the university experimental forest “Bois de Lauzelle” (part of Natura 2000 site “Vallée de la Dyle à Ottignies”; Ottignies, Wallonia; 50.680434 N, 4.614885 E; Figure 3). *Limenitis camilla* and its host plants *L. periclymenum* are both found in Bois de Lauzelle. Healthy and fully developed *L. periclymenum* (*L. xylosteum* was not considered since it was not found in any sampling areas) used as host plant during behavioural experiments were collected near the flight arenas, re-planted in a textile pot, and placed in one of the four tunnels of the experimental cage defined as the “target tunnel”. The other three arms were left empty as “control tunnels”. The identities of target and control tunnels were kept constant across repeated trials for each butterfly. A temperature and humidity sensor (HOBO U23 Pro v2 Temperature/Relative Humidity Data Logger, Onset, MA, USA) was attached to the ceiling at the end of each tunnel to acquire data on microclimatic variation between tunnels (recorded every 5 min for the whole study duration).

Before behavioural trials, butterflies were sensitised to lower their threshold to oviposition behaviour during following behavioural trials. Butterflies were released and presented with the host plant for 10 min in a 2 × 3 × 2 m temporary enclosure built at the end of one of the flight tunnels. Butterflies that did not oviposit during a sensitisation trial were tested again the following day, whereas butterflies which oviposited were considered “sensitised” and used later the same day for behavioural trials.

Behavioural trials were carried out in the whole experimental arena (Figure 3). Butterflies were brought to the central part of the arena inside plastic boxes whose cover was loosened to be removed with a long stick from an operator outside the arena. A behavioural trial started when the individual flew out from the box and lasted for 30 min; during this time, butterfly behaviours and movements were voice-recorded. Trials were stopped immediately upon oviposition. Leaves on which butterflies oviposited were removed to avoid biased behaviour in successive trials as eggs are known to be used as social cues by some butterfly species. Behavioural trials were conducted on each butterfly over successive days, until death or when wing wear did not allow flight.

During experimental trials, we monitored and voice-recorded all butterfly behaviours, which were subsequently pooled into three behavioural categories:

- “Departing”: flight activities associated with either butterflies repeatedly flying into the covering net of the tunnels or with flight towards the sun, a known tendency in butterflies. We interpreted these behaviours as the initiation of a dispersal movement from one patch of potential habitat (in our case, a patch inside the flight structure) to another [54,55]. We considered increased time spent in departing flight in butterflies as an indication of an increased investment in dispersal behaviour.
- “Navigation”: all behaviours involving interactions with the habitat inside the flight tunnels: flying freely in petal-like patterns, over/around the host plant, landing on the host plant, walking on the tunnel structure or on the ground (also defined “routine movements”, see [56]).
- “Resting”: sitting still on the net, on the tunnel structure or on the ground.

We aggregated the durations (in seconds) of each behaviour category per individual, tunnel and experimental trial. Each behaviour was associated with tunnel-location of the host plant, average temperature, humidity and insolation, which were recorded during the experiments and averaged over each 30 min trial. We used Generalised Linear Mixed Models (GLMMs) with a negative-binomial link function to test whether there were differences in the total duration of the various behavioural categories. We considered trial and butterfly ID as random factors whereas behavioural category, temperature, humidity and insolation were considered regression covariates. We next tested whether the presence of the host plant in a tunnel was associated with a different residence time in the tunnels. We further aggregated the dataset using the presence/absence of host plant in the tunnels as a grouping factor, thus discarding tunnel IDs. This dataset was used to model the duration of behavioural categories by adding an interaction term between target (with host plant) and control (without host plant) tunnels and behavioural category to the model. Temperature, humidity and insolation were used as additional covariates. An offset term was added to this latter model to account for control tunnels having three-times higher probability of hosting behavioural activity with respect to the sole target tunnel. The non-aggregated dataset was used to fit a third model to test for differences in behaviour between butterflies with different origin, i.e., fragmented vs. homogenous woodlands. This model included an interaction term between behavioural category and origin of the butterfly. We visually inspected models’ residuals and used the Akaike Information Criterion (AIC) to select all the final models. All the analyses were carried out in R 3.6.3 (R Core Team 2020) through the functions available in lme4 package [57]. The dataset and R code to reproduce all model results are available at https://osf.io/2ej7x/ (accessed on 10 August 2023).

## 3. Results

### 3.1. Selection of Habitats with Low and High Levels of Functional Connectivity within Areas of Frequent L. camilla Observations (Expectation 1, E1)

The number of aggregated *L. camilla* observations per 5 km cell (henceforth abundance) ranged from 1 to 897 (Figure 1). The highest abundance was found in south-west Wallonia (Namur province) and in the detached Comines-Warneton municipality (Hainaut province). The north, north-east and south-east parts of Wallonia lacked almost entirely observations for *L. camilla* in the past 40 years. The maximum number of years and decades with observations for a cell were, respectively, 26 and 5, with a spatial distribution similar to that shown by *L. camilla* abundance data (Figure 1).

**Figure 4:**
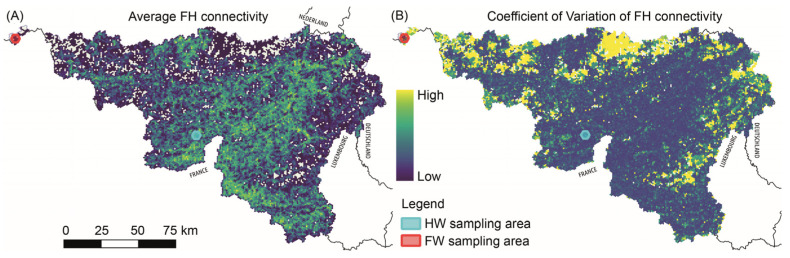
Maps reporting: (**A**) the average functional habitat (FH) connectivity and (**B**) its coefficient of variation (right) for Wallonia. Transparent cells inside Wallonia do not contain FH. The red and light-blue small hexagons overlaid on the maps show homogenous (HW) and fragmented (FW) woodlands from where butterflies were collected.

Stable functional woodland habitats were mainly distributed along a diagonal line stretching from the south-east of Namur province to the north-east of Liege province (Figure 4). The connectivity analysis showed that regions with abundant highly connected habitats underwent only moderate changes when compared to areas with lower habitat connectivity, such as north-west Hainaut, west Walloon Brabant and south-east Liege (Figure 4). These latter areas were characterised by a higher temporal variability (i.e., loss) of functional woodland habitat cover and connectivity. Further, we selected the two 5 km cells (see Figure 1) having the lowest and highest average connectivity values, respectively, among cells with the highest *L. camilla* abundance and persistence. These cells represented reference sampling locations in fragmented and homogenous woodlands.

**Figure 5:**
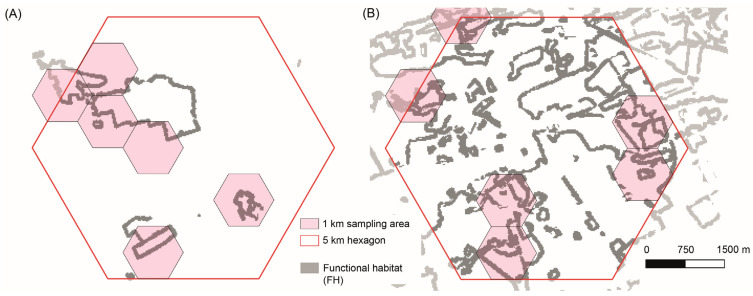
Sampling areas: (**A**) Comine-Warneton cells (fragmented woodlands) and (**B**) Doische (homogeneous woodlands). The red cells with canvas pattern were 1 km cells chosen for collecting *L. camilla* and for quantifying host plant abundance. The background map reports in gray the extent of functional habitat.

The cell with the lowest connectivity values (i.e., area of functional habitat available to a butterfly that is able to fly 500 m over the landscape matrix) was located in the detached enclave of Comines-Warneton (red hexagon in Figure 4). This area was characterised by very low functional habitat connectivity (average 0.2 km^2^), while showing moderate *L. camilla* abundance: 342 individuals observed in 11 different years in the last five decades. We further verified that the low connectivity of this detached enclave was not due to an “edge effect” by using land cover additional datasets for both Flanders and France (see Supplementary Materials, Figure S1 for further information). The cell with the highest connectivity (average 91 km^2^) values was centred on the village of Doische. This cell also contained a high number of *L. camilla* observations, 491, collected in 21 years over three decades (light-blue hexagon in Figure 4). Inside these two 5 km cells, we selected the 1 km cells with the highest cover of forest functional habitat (Figure 5). Inside and in the proximity of these 1 km areas, we collected *L. camilla* and assessed the abundance of their host plant species.

### 3.2. Spatial Distribution and Abundance of the Host Plant (Expectation 2, E2)

We carried out a total of 10 host plant surveys, six in homogenous woodlands and four in fragmented ones and found *L. periclymenum* in eight out of the 10 total surveys, while *L. xylosteum* was not found in any areas. We fitted a Linear Mixed Model (LMM) considering plot ID as a random factor to test for differences in the number of *L. periclymenum* stems between the two areas. Model results showed that the sampled areas in fragmented habitats had an overall higher abundance of the host plants with respect to homogenous habitat areas (LMM; *β =* 6.25, *df* = 34.45, *p* = 0.015). On the contrary, the Shannon index calculated at survey quadrant level was higher in homogenous habitats (H = 2.23, sd = 0.19) suggesting that *L. periclymenum* was more evenly distributed in these areas than in fragmented habitats (H = 1.83, sd = 0.28).

### 3.3. Behavioural Activity of Populations from Fragmented vs. Homogenous Woodlands (Expectations 3 and 4, E3 and E4)

**Figure 6:**
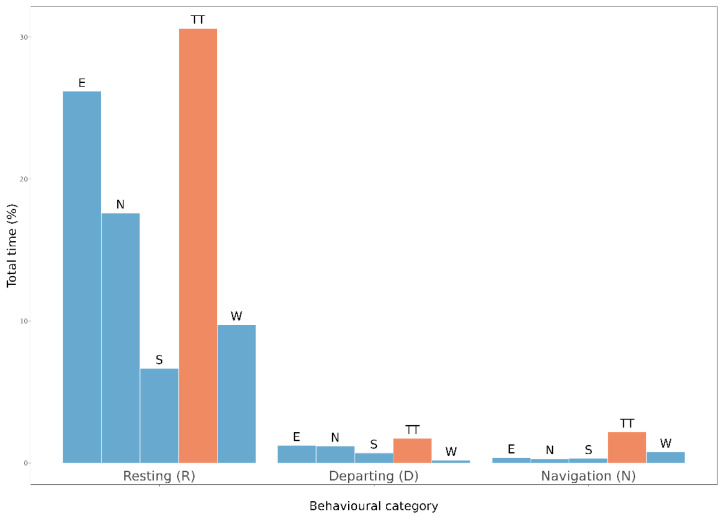
Barplot showing the percentage of total trial time (for all 14 tested butterflies) spent in the different behavioural categories. Red bars indicate time spent in the target tunnel (TT), whereas blue bars indicate control tunnels (which can be any of the four tunnels named following their long-axis cardinal direction).

We collected a total of 46 *L. camilla* (from 29 June 2021 to 11 August 2021), 40 from homogenous and six from fragmented woodlands, and 36 underwent one to six 10 min sensitisation tests. During a total 71 sensitisation tests, 10 butterflies laid eggs, seven from homogenous and three from fragmented habitats (representing 17% and 50% of the two groups, respectively), during 11 different tests. Twenty-six 30 min behavioural trials were carried out on 10 butterflies from homogenous and four from fragmented woodlands, which oviposited during sensitisation or underwent at least three sensitisation tests. Only one *L. camilla* (from a homogenous woodland) oviposited (twice) during behavioural trials. Butterflies spent more time resting (R = 90.9%, of which 1.1% was represented by walking) compared to departing and navigation behaviours, which represented 5.1 and 4.0% of the total recording time, respectively (Figure 6). We further assessed linear combinations of model covariates, which revealed how resting periods were significantly higher than both departing and navigation flights, whereas time spent in these latter categories did not differ between them (μ = −0.552, *p*-value = 0.66). In addition, butterflies spent significantly more time resting, and tended to exhibit less departing and more navigation in the target tunnel with respect to the other tunnels (Table 1).

Overall, butterflies spent considerably more time in the east and south tunnels (212 and 177 min, respectively), which had higher average temperature during trials (25.5 and 23.9 °C, respectively) than in north and west tunnels (113 and 60 min, respectively), which recorded lower average temperatures (23.3 and 22.4 °C). Despite these results, temperature, humidity and insolation showed all very small, non-significant, model coefficients and did not change model AICs; thus, they were removed from all final models.

**Figure 7:**
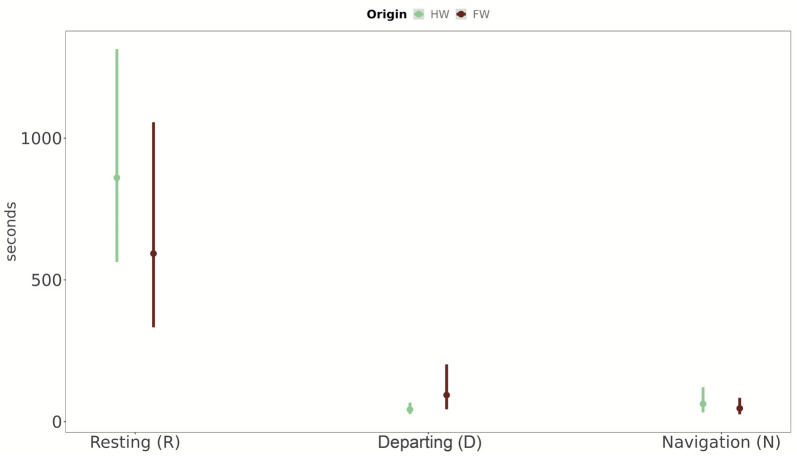
Variation in three main behavioural categories (resting, departing and navigation) for homogeneous (in green), and fragmented (in brown) woodlands, with model marginal means and 95% CIs. The 95% CIs have been calculated considering only model regression parameters (i.e., not considering the uncertainty in the model variance parameters).

The origin of the tested butterflies did not affect the time they spent resting. On the contrary, the interaction term suggested that butterflies originating from fragmented woodlands spent more time in departing flight with respect to butterflies that originated from homogeneous habitats. Navigation activities did not vary between habitats of origin (Table 2, Figure 7).

## 4. Discussion

Habitat fragmentation is transforming Earth’s ecosystems and contributing to the ongoing biodiversity crisis [58]. Species depending on specific habitats or resources are more affected by fragmentation due to their lower competition capacity in more isolated and reduced habitats [59]. Among the most intriguing and less studied consequences of habitat fragmentation on biota is their behavioural response, whose phenotypic plasticity can affect the evolutionary trajectories of species [16,60,61].

Here, we conducted a pilot study in which we addressed whether habitat fragmentation has an effect on behavioural strategies related to oviposition in populations of the woodland specialist butterfly *L. camilla*. We first discussed our results on the connectivity of the functional habitat of *L. camilla* in Wallonia (E1 and E2) and then deepened the comparison between behavioural strategies of populations originating from homogenous and fragmented woodlands (E3 and E4). We discussed the limitations of this pilot study in a final paragraph, where we advised caution in the interpretation of our results due to the limited sample size, while suggesting ways forward to overcome them.

### 4.1. Functional Habitat Connectivity and Host Plant Spatial Distribution (E1 and E2)

We found areas in Wallonia with opposite trends in the connectivity of the functional habitat of *L. camilla*, represented by secondary forests characterised by open sunny areas in the tree vegetation cover [10,62]. Western Europe suffered massive forest loss starting from late prehistory and antiquity, followed by a reforestation phase and renewed deforestation in medieval times, with a dramatic acceleration during modern times [63,64,65]. These long-lasting land-cover changes often caused extreme fragmentation of remnant forest habitats, but areas with a more complex topography remained less exploited due to higher costs of land transformation [66]. South-western areas of Wallonia are characterised by larger “conserved” woodlands interrupted by smaller agricultural lands; here, we found that the distance between woodland patches is often well into the average dispersal capacity of *L. camilla,* and thus these woodlands form an extended network of connected functional habitats. In addition, in homogenous woodlands, *L. camilla* host plants were less abundant but more evenly distributed in space, producing less isolated and more regularly spaced oviposition habitats. In contrast, the fragmented woodlands are mainly present in the (less topographically complex) north and east boundary portions of Wallonia, where host plants were overall more abundant but also more isolated (i.e., aggregated). These differences in host plant spatial distribution in our study area may be explained by the autoecology of *L. periclymenum*, which prefers shady edge habitats where roots stay in the shade and shoots can climb up more easily to the sun. In Wallonia, fragmented patches of forest often present sharp edges shared with agricultural or urban lands where *L. periclymenum* could become dominant. On the contrary, more extended and connected woodlands are characterised by frequent but less sharp woodland edges, which may explain an even spatial distribution of *L. periclymenum* in these areas [46].

### 4.2. Behavioural Strategies of Populations from Fragmented vs. Homogenous Woodlands (E3 and E4)

Behavioural trials showed that *L. camilla* females remained spending more time in tunnels with host plants, regardless of the habitat of origin (confirming hypothesis E3). In addition, individuals from fragmented and isolated habitats spent more time departing than individuals originating from connected functional habitats (confirming E4). Plastic behavioural responses related to flight and specifically oviposition site selection are fundamental for specialist butterflies that experience environments where their host plants (being limited) are more likely to be infrequent (coarse-grained environmental variation [61]). The observed increased investment in departing movements may be therefore due to a positive selective pressure in butterfly populations from more isolated habitats. When faced with an environment where oviposition resources are limited to isolated small patches of functional habitat, butterflies may readapt to local conditions, for example, exploiting a sub-optimal host plant, or they may disperse in search of more suitable habitats (see [67]). A stronger investment in departing flights, which for example may result in movements from one potential habitat patch to another, observed during the behavioural trials, may be a response to the perception of a non-suitable habitat in order to increasing the likelihood of finding a new (disconnected) suitable habitat. Hence, we interpreted an increased time spent in departing flight in butterflies from fragmented woodlands as an indication of an increased investment in dispersal behaviour. However, our experimental settings prevented us from determining the ending point of departing flights. We cannot rule out that this type of flight is not necessarily associated with emigration in a different habitat patch and not with a more efficient strategy to exploit infrequent resources inside the same habitat patch. In line with this hypothesis, we found that host plants were both more abundant and more aggregated in space in fragmented woodlands, producing more disconnected oviposition habitats than in homogenous woodlands. The higher investment of butterflies from fragmented habitats in departing flight may be adaptive, as it may help them find more isolated, but locally more abundant clusters of host plants in the same woodland patch (i.e., increasing the range of local environments experienced [61]). In this regard, a study on the effect of plant spacing on the movement of flea beetles found that less evenly distributed plant patches were associated with more random foraging patterns than when plants were more evenly distributed in space [68].

Our findings add to a body of work on butterfly behaviour and conservation showing that butterflies occupying small, fragmented habitat patches display phenotypic changes, including changed mass allocation to flight muscles and to reproduction [33,34,37,67,69,70,71], compared to populations occupying more contiguous patches. Our findings did not support the navigation assumption of hypothesis E4, despite the non-significant increased time spent “navigating” by butterflies from connected habitats (see Figure 7). This result may be related to the short time that butterflies spent “navigating” during the behavioural trials, possibly due to the experimental conditions, as discussed in the following section.

### 4.3. Limitations and Perspectives

We used a large experimental setup placed in a habitat specific to *L. camilla* to achieve conditions ecologically relevant for behavioural studies in insects [60]. This approach allowed us to study *L. camilla* behaviours in conditions as close as possible to their natural environment. Such experiments cannot indeed be easily carried out for highly vagile species under full field conditions (but see [72]) and tracking devices are not yet light enough for experiments with small and medium-sized butterflies, including most European butterflies [73,74,75]. We also faced practical complications that limited the sample size of tested butterflies. We were unable to test all the collected individuals due to colder and wetter than usual weather conditions (for more information see the Supplementary Materials, Figure S2). Moreover, we tested butterflies in an outdoor arena, which, despite being much larger than common laboratory behavioural cages, may have not been spacious enough for wild butterflies. We conceived our setup as a cross-shaped structure that had the advantage of mimicking behavioural bioassays of four-way olfactometers used to study animal behaviour in laboratory conditions [76], and that had been also previously tested on another butterfly species [42]. This type of bioassay is very flexible and allows for experiment reproducibility, but it has a low volume–perimeter ratio and thus a higher chance that tested individuals contact the covering net, perceiving it as a threat or impediment, which may not be suited for highly vagile flying species. Despite some limitations, our results provide a rare quantification of behavioural strategies used by wild-caught insects tested in outdoor conditions, making them, and the guidelines for conservation that can be inferred from them, precious and worth disseminating especially considering the aggravating insect crisis.

## Figures and Tables

**Table 1:** Summary table for a model testing time spent by butterflies in the target tunnel (TT, i.e., tunnel with the host plant) in the three different behavioural categories, by also considering the interaction between tunnel type and behavioural category. Random factors: σ trial = 3.7 × 10^−6^; σ id = 6.5 × 10^−7^. D = departing, N = navigation.

Model Term	Estimate	Std. Error	z Value	Pr (>|z|)
*Intercept*	3.62	0.21	17.46	<0.001
*Tunnel TT*	2.05	0.36	5.94	<0.001
*Departing (D)*	−2.58	0.33	−8.32	<0.001
*Navigation (N)*	−2.61	0.41	−5.85	<0.001
*TT:D*	−0.12	0.55	−0.43	0.831
*TT:N*	0.74	0.67	0.71	0.262

**Table 2:** Modelled time spent in the three different behavioural categories according to the origin of the butterfly (FW = fragmented woodlands; D = departing, N = navigation). Summary table with random factors: σ trial = 1.51 × 10^−6^; σ id = 6.8 × 10^−7^, σ tunnel = 3.03 × 10^−6^.

Model Term	Estimate	Std. Error	z Value	Pr (>|z|)
*Intercept*	6.76	0.22	31.26	<0.001
*Origin FW*	−0.37	0.36	−1.02	0.311
*Departing (D)*	−3.00	0.32	−9.47	<0.001
*Navigation* (N)	−2.61	0.40	−6.57	<0.001
*Origin FW:Departing*	1.16	0.58	1.98	0.043
*Origin FW:Navigation*	0.08	0.58	0.14	0.892

## Data Availability

The dataset and R code to reproduce all model results are available at https://osf.io/2ej7x/ (accessed on 10 August 2023).

## Supplementary Materials

The following supporting information can be downloaded at: https://www.mdpi.com/article/10.3390/insects14090737/s1. Figure S1. The reference sampling areas for the fragmented woodland habitat (FW) are shown in as red hexagons. The land use category equivalent to “broadleaved forest” is reported in the background (grey) as derived from three different land cover standard datasets: “Boswijzer Vlaanderen 2019”, “Ecotope 2019” and “Theia Land Cover Map 2018”. Figure S2. Barplots comparing temperature and precipitation for the year 2021 (light grey) versus the 2000-2020 (dark grey) period. The monthly standard deviation is indicated by dashed error bars. The two vertical red lines delimit start and the end months of experimental trials).
