# Supplementary material for: The Effect of Habitat on Insect Movements: Experimental Evidence from Wild-Caught Butterflies
Source: Insects. 2023 Aug 31;14(9):737. doi: 10.3390/insects14090737 (PMC10531938; doi:10.3390/insects14090737)
Supplement: Supplementary file 1 [file insects-14-00737-s001.zip › insects-2579495-supplementary.pdf]

The effect of habitat on insect movements: experimental evidence from wild-caught butterflies

Matteo Marcantonio [1,*,†], Raluca Voda [1,*,†], Daniele Da Re [2], Quentin Igot [2], Roger L. H. Dennis [3], Aurélien Vielfaure [1,4], Sophie O. Vanwambeke [2] and Caroline M. Nieberding [1]

1 Earth & Life Institute, University of Louvain (UCLouvain), Carnoy building, Croix du sud 4-5,1348 Louvain-la-Neuve, Belgium; vielfaure.aurelien@gmail.com (A.V.); caroline.nieberding@uclouvain.be (C.M.N.)
2 Georges Lemaître Centre for Earth and Climate Research, Earth & Life Institute, University of Louvain (UCLouvain), Place Louis Pasteur 3, Bâtiment Mercator, 1348 Louvain-la-Neuve, Belgium; daniele.dare@uclouvain.be (D.D.R.); quentin.igot@gmail.com (Q.I.); sophie.vanwambeke@uclouvain.be (S.O.V.)
3 UK Centre for Ecology & Hydrology, Maclean Building, Benson Lane, Wallingford OX10 8BB, UK; rlhdennis@aol.com
4 L'Institut Agro Dijon, 26, bd Docteur Petitjean-BP 87999, 21079 Dijon Cedex, France

* Correspondence: matteo.marcantonio@uclouvain.be (M.M.); raluvoda@gmail.com (R.V.)
† These authors contributed equally to this work.

Supplementary Material 1

The reference sampling area for fragmented habitats (FW) was in the municipality of Comine-Warneton, which lays between the Walloon and Flemish regions of Belgium, and France. We used only data from Wallonia to derive FH for *L. camilla,* due to important differences in the availability of historical land cover data for surrounding areas. The presence of large swaths of FH outside Wallonia could have undermined our methodological approach, thus, we decided to consider current land use data to confirm that the FW sampling area was disconnected from large functional habitat patches also outside Wallonia. For this scope, we considered two additional standard land cover maps: "Boswijzer Vlaanderen 2019" for Flanders (http://www.geopunt.be/) and "Theia Land Cover Map 2018" for France (https://www.theia-land.fr/en/product/land-cover-map/). We first extracted the land use class "Broadleaved forest"

from these two layers and then fused it with the Walloon equivalent "Ecotope 2018" as reported in the map shown in figure SM1. This figure highlights that patches of potential FH outside Wallonia were very small and separated by several kilometres or matrix.

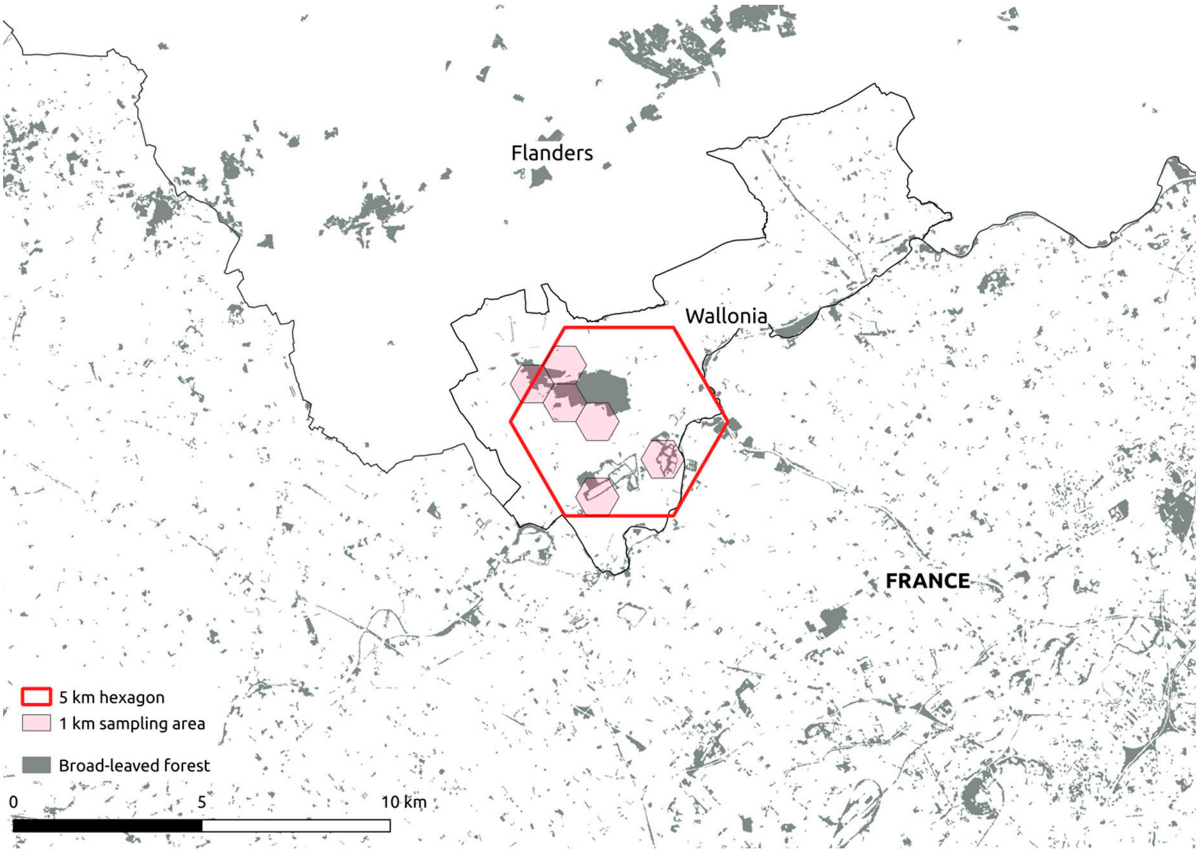

Figure S1. The reference sampling areas for the fragmented woodland habitat (FW) are shown in as red hexagons. The land use category equivalent to "broadleaved forest" is reported in the background (grey) as derived from three different land cover standard datasets: "Boswijzer Vlaanderen 2019", "Ecotope 2019" and "Theia Land Cover Map 2018".

Supplementary Material 2

We derived monthly cumulative precipitation and monthly average temperatures and standard deviations for the 2000-2020 period as well as for the year 2021 from the weather station ("Brussels South": 50.459 N, 004.454 E) closest to the locations of the experimental tunnel available from the Global Surface Summary of the Day datasets provided by the National Oceanic and Atmospheric Administration (NOAA GSOD: https://www.kaggle.com/noaa/gsod,

accessed on 03/09/2022). We then compared monthly precipitation and temperature between these two periods in the barplot shown in figure SM2. The figure shows how temperature and precipitation for the year 2021 were often well below and well above respectively, the 2000-2021 long term averages from April to August (period of experimental trials).

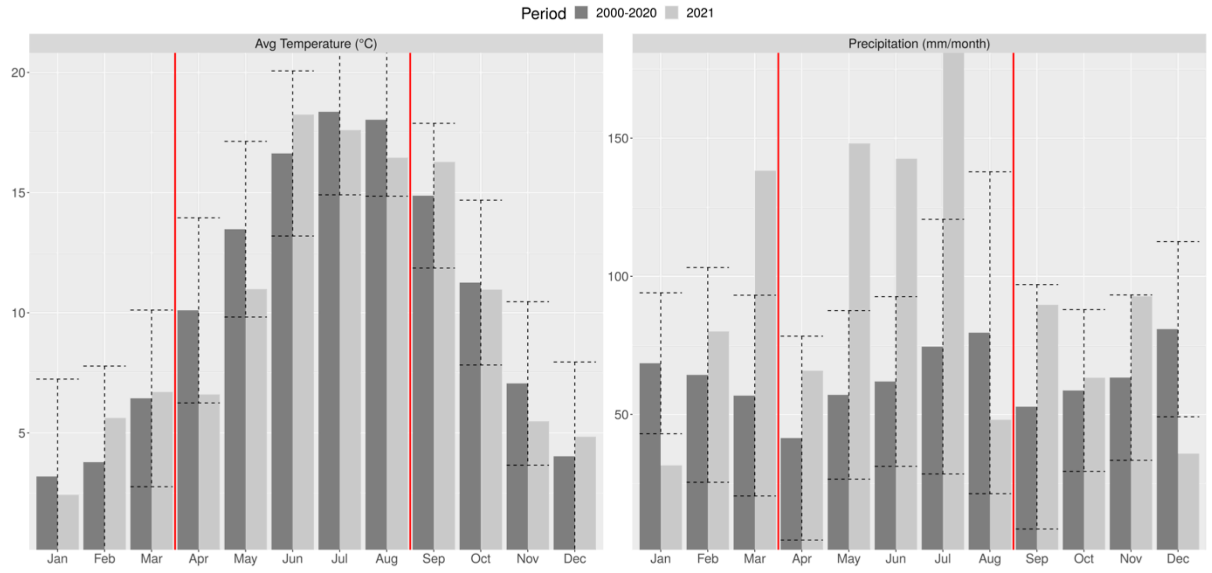

Figure S2. Barplots comparing temperature and precipitation for the year 2021 (light grey) versus the 2000-2020 (dark grey) period. The monthly standard deviation is indicated by dashed error bars. The two vertical red lines delimit start and the end months of experimental trials.
